# Neuronal plasma biomarkers in acute ischemic stroke

**DOI:** 10.1177/0271678X241293537

**Published:** 2024-10-25

**Authors:** Julia K Gundersen, Fernando Gonzalez-Ortiz, Thomas Karikari, Bjørn-Eivind Kirsebom, Katrin Mertes, Henrik Zetterberg, Hlin Kvartsberg, Ole Morten Rønning, Berglind Gísladóttir, Kaj Blennow, Tormod Fladby

**Affiliations:** 1 Department of Neurology, 60483 Akershus University Hospital, Lørenskog, Norway; 2 Department of Physiology, Institute of Basic Medical Sciences, University of Oslo, Norway; 3 Department of Psychiatry and Neurochemistry, Institute of Neuroscience and Physiology, the Sahlgrenska Academy at the University of Gothenburg, Mölndal, Sweden; 4 Clinical Neurochemistry Laboratory, 56749 Sahlgrenska University Hospital, Mölndal, Sweden; 5 Department of Psychiatry, University of Pittsburgh, Pittsburgh, PA, USA; 6 Faculty of Medicine, 9174 University of Freiburg, Freiburg, Germany; 7 Department of Neurodegenerative Disease, UCL Institute of Neurology, Queen Square, London, UK; 8 UK Dementia Research Institute at UCL, London, UK; 9 Hong Kong Center for Neurodegenerative Diseases, Clear Water Bay, Hong Kong, China; 10 Wisconsin Alzheimer’s Disease Research Center, University of Wisconsin School of Medicine and Public Health, University of Wisconsin-Madison, Madison, WI, USA; 11 Institute of Clinical Medicine, Campus Ahus, University of Oslo, Norway; 12 Clinical Molecular Biology (EpiGen), Medical Division, Akershus University Hospital and University of Oslo, Norway

**Keywords:** Stroke, acute ischemic stroke, tau, MRI, brain-derived tau

## Abstract

Early imaging-based detection of acute ischemic stroke (AIS) has improved in the era of reperfusion therapy. Despite of this, prognosis of outcome after AIS remains a challenge. Therefore, parameters that support clinical decision making are sought. Blood-based biomarkers have the potential to provide valuable information in addition to the established prognostic factors. Neuronal biomarkers of acute or degenerative neuronal injury have shown to be reliably detected in plasma. These biomarkers are well-established in neurodegenerative pathology, such as Alzheimer’s disease. In this study, we explored the association between stroke diameter and plasma biomarkers for neuronal injury and tau pathophysiology (brain-derived tau [BD-tau], phosphorylated-tau-217 [p-tau21] and neurofilament light [NfL]) in patients (n = 193) admitted to the acute ward, Akershus University Hospital. All patients received a final diagnosis of AIS, transient ischemic attack or stroke mimics. Blood samples were obtained the day after admission. We find that levels of BD-tau (p = .004) and NfL (p = .011) were higher after AIS than in patients with stroke mimics. The cortical stroke diameter correlated with BD-tau (tau-b = 0.64, p < .001) and p-tau217 (tau-b = 0.36, p = .003). Linear regression confirmed BD-tau to be the strongest variable associated with stroke diameter, pointing to the potential clinical value of plasma BD-tau in outcome prediction after AIS.

## Introduction

Acute ischemic stroke (AIS), a leading cause of mortality and long-term disability, poses an enormous global burden.^1,2^ Thrombolysis and thrombectomy are highly effective but time-sensitive treatments that improve long-term outcome after AIS.^3^ Approximately 25% of patients presenting with AIS have a non-ischemic neurological or non-neurological condition, such as peripheral vestibular dysfunction (23.2%), seizures (13.0%) functional disorders (9.7%) and migraine (7.8%).^4^ Differentiating these so-called stroke mimics from true AIS is important to reduce unnecessary examination and treatment this patient group.^5^ Furthermore, clinical outcome, prognosis and need for rehabilitation may be difficult to establish in the first days after AIS. Therefore, blood-based biomarkers reflecting neuronal injury are sought to supplement radiological examination and aid in clinical decision making.^6,7^

Tau, a microtubule-associated intracellular protein located in neuronal axons, has emerged as a potential biomarkers for AIS,^8^ demonstrating a significant correlation with the infarct size.^9^ Phosphorylation of tau is regulated in the developing foetal brain^10,11^ into adulthood, where it plays a physiological role in the assembly and stabilisation of axonal microtubule as well as synaptic structure.^12–14^ Tau hyperphosphorylation is recognised as a hallmark of Alzheimer disease (AD) as part of neurofibrillary tangles.^13,15^

Most commonly, quantification of tau protein is performed in cerebrospinal fluid (CSF), which is unfeasible in the acute setting of AIS. Therefore, methods for quantifying tau in plasma, which is in equilibrium with CSF,^16^ have been developed. However, different tau isoforms are also abundant in the peripheral nervous system.^13^ Recently, we published novel findings on brain-derived tau (BD-tau), a specific splice variant of tau encompassing only tau from the central nervous system,^17^ thus allowing quantification without disturbance from the periphery. BD-tau has demonstrated to be a promising marker of neurodegeneration in AD^17^ and a severity indicator in traumatic brain injury.^18^ A recent study have demonstrated BD-tau to be a potential marker of functional outcome after AIS,^19^ however validation across multiple cohorts is still pending.

In this study, we aimed to quantify BD-tau, isoform of phosphorylated-tau-217 (p-tau217) and neurofilament light (NfL, an established neuronal injury marker) in patients presenting with stroke mimic, transient ischemic attack (TIA) or AIS. We hypothesised that patients with AIS would have higher concentration of BD-tau in plasma compared to patients with stroke mimics. Moreover, we hypothesised that BD-tau concentration in plasma would be positively correlated with the infarct size as measured on in-patient magnetic resonance imaging (MRI).

## Methods

### Study population and data collection

We analysed a prospective cohort of 193 patients admitted to the stroke unit at Akershus University Hospital in Oslo, Norway, between 06/06/2015–31/01/2018. The study was approved by the Regional Committees for Medical and Health Research Ethics (ID: 579301), based on ethical guidelines of the Declaration of Helsinki. All recruited patients provided written consent. Patient data were anonymised and collected from in-hospital patient records. We collected patient characteristics and risk factors of cerebrovascular disease such as sex, age, medical history of AIS, TIA, ischemic heart disease, diabetes, atrial fibrillation, hypertension, hypercholesterolemia and current smoking. Venous blood samples were obtained at admission for analysis of haemoglobin, platelets, leukocytes, c-reactive protein (CRP), creatinine, estimated glomerular filtration rate (eGFR), alanine aminotransferase (ALT), aspartate aminotransferase (AST), glucose, sodium, and potassium. Additionally, blood pressure and temperature at admission were collected. Upon admission, patients underwent a clinical examination and neurological deficits were measured with the National Institutes of Health Stroke Scale (NIHSS).^20^ Cerebral CT was routinely performed within 15 minutes of admittance. A final diagnosis of AIS, TIA or mimic was established based on comprehensive clinical assessment and findings on imaging scans (CT and/or MRI). Clinical management followed the Norwegian National guidelines for AIS and TIA.^21^ Patients with suspected or diagnosed AIS were scheduled for an inpatient MRI scan prior to discharge (n = 134). The MRI modalities included at least a T1-weighted image, a T2-weighted image, diffusion weighted imaging (DWI), and FLAIR. MRIs were assessed for stroke location and diameter (OMR), and presence of chronic ischemic white matter lesions (WML; JKG) by investigators blinded to relevant clinical information. DWI sequences were used to distinguish between acute and old ischemic lesions. Axial single-plane measurements of stroke diameter were performed on DWI sequences. WML was assessed in deep and periventricular regions separately with Fazekas scale,^13^ of which the mean value is presented. The investigators were blinded to the levels of biomarkers and other clinical severity markers.

### Biomarker quantification

Venous EDTA blood samples (Vacuette K3EDTA, G454021) for quantification of stroke biomarkers were obtained the day after admission. The tubes were centrifuged within 2 hours at 2000 g for 10 minutes at room temperature, followed by aliquoting and freezing directly at −80°C. Plasma BD-tau and p-tau217^22^ measurements were performed on the Simoa HDX (Quanterix, Billerica, MA). Analytical validation followed protocols described previously.^17^ These measurements were performed at the Clinical Neurochemistry Laboratory, Sahlgrenska University Hospital, Mölndal, Sweden. Plasma NfL were measured in a R-plex format using Human Neurofilament L Assay (K1517XR-2) by the QuickPlex SQ 120 system from Meso Scale Discovery (MSD, Rockville, MD, USA). The NfL analyses were carried out according to the manufacturers’ procedures. All samples were analysed in duplicates and quality control samples with RD threshold of 15% assured inter-plate and inter-day variation. The NfL analyses were performed at the Section of Clinical Molecular Biology (EpiGen) at Akershus University Hospital.

### Statistics

Statistical analyses were performed in R (v.4.3.0). For two-sample analysis, comparing mimic versus TIA and mimic versus AIS, Kruskal-Wallis test with Dunn’s correction was applied. Fisher’s exact test was used to explore associations between categorical variables. Linear correlations were examined using the Kendall’s tau-b correlation coefficient (tau-b). Backwards stepwise linear regression analysis was used to explore the association between stroke diameter and potential explanatory variables. Plasma BD-tau, p-tau217 and NfL were log-transformed prior to analyses. A full model with stroke diameter as the dependent variable was fitted with the following independent variables: log-transformed BD-tau, log-transformed p-tau217, log-transformed NfL, NIHSS score at admission, age (years), medical history of previous TIA or stroke, eGFR, Fazekas scale score and number of risk-modifying drugs. Non-significant independent variables were then iteratively excluded using stepwise elimination guided by the lowest partial R-squared, while controlling for decreasing Bayesian Information Criterion (BIC). Stepwise removal was repeated until only significant independent variables remained in the model.

## Results

### Study population

Among 193 admitted patients, 102 were diagnosed with acute stroke. Two patients initially diagnosed with TIA were later reassessed as AIS based on MRI findings. The median (IQR) duration from ictus to admission was 9.5 hours (2.0–35.0). Nineteen (18.6%) AIS patients were treated with intravenous thrombolysis and 3 (2.9%) underwent thrombectomy. In comparison, 60 patients received a diagnosis of TIA. Self-reported TIA duration was 45 minutes (15–120). There were 31 patients with suspected AIS who received a final diagnosis of “stroke mimic”.

### Plasma BD-tau and NfL are increased in AIS patients

**Figure 1.**
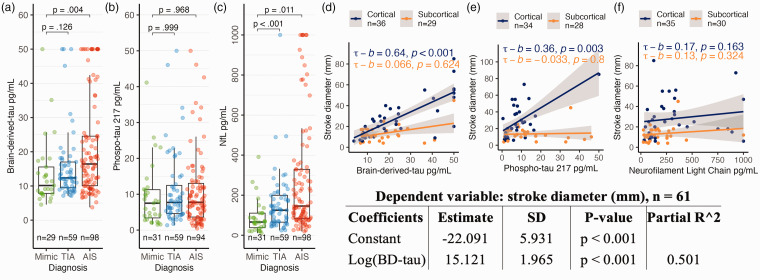
Graphs (a)–(c) shows the distribution of (a) BD-tau, (b) p-tau217, and (c) NfL stratified by diagnosis. Graphs (d)–(f) show the linear correlation between stroke diameter measured in mm on MRI, and values of (d) BD-tau, (e) p-tau217 (f) NfL. The final significant iteration of the backwards stepwise linear regression analysis is shown in the panel below, where log-transformed BD-tau was the only significant variable associated with stroke diameter (dependent variable).

Median duration between reported ictus and blood sampling for biomarker analysis was 26.5 hours (21.6–35.1) for TIA and 40.5 hours (25.8–72.5) for AIS patients. BD-tau (p = .004) and NfL (p < .011) levels were significantly elevated in AIS patients as compared to mimics (Figure 1). Likewise, NfL (p < .001) was greater in TIA than mimic patients. P-tau217 did not differentiate between the groups.

### Tau markers and NIHSS score

AIS patients had significantly higher NIHSS scores at admission and on the subsequent day (Table 1, *supplementary figure 1,* p = .050) as compared to mimics. Among all the biomarkers assessed, only p-tau217 exhibited some correlation with NIHSS score in AIS at admission (tau-b = .17, p < .050). BD-tau demonstrated the second strongest correlation with NIHSS score at admission, albeit not statistically significant (tau-b = .14, p = .066). None of the biomarkers demonstrated significant correlation with NIHSS score on day 2.

**Table 1.** Patient characteristics, stratified by final diagnosis of stroke mimic (n = 31), TIA (n = 60) and AIS (n = 102).

								Fisher’s exact	Kruskal Wallis-Dunn	
Patient characteristics		Mimic n = 31		TIA n = 60		AIS n = 102			*TIA*	*AIS*
*Age (years)*		62.0	[56.5, 71.0]	74.5	[65.0, 84.0]	72.0	[61.0, 78.0]		.003*	.049*
*Sex, n (%)*	*Female*	17	(54.8)	36	(60)	37	(36.3)	.009*		
Risk factors										
*Smoking, n (%)*	*Yes*	7	(22.6)	11	(18.3)	26	(25.5)	.584		
*Cognitive impairment, n (%)*	*Yes*	3	(9.7)	1	(1.7)	2	(2)	.090		
*Previous TIA or stroke, n (%)*	*TIA*	3	(9.7)	9	(15)	5	(4.9)	.165		
	*Stroke*	2	(6.5)	9	(15)	15	(14.7)			
*Ischemic heart disease, n (%)*	*Yes*	1	(3.2)	11	(18.3)	16	(15.7)	.179		
*Number risk-modifying drugs, n (%)*	*0*	9	(29.0)	15	(25.0)	29	(28.4)	.302		
	*1*	11	(35.5)	8	(13.3)	20	(19.6)			
	*2*	7	(22.6)	14	(23.3)	25	(24.5)			
	*3*	3	(9.7)	18	(30.0)	18	(17.6)			
	*4*	1	(3.2)	4	(6.7)	9	(8.8)			
	*5*	0	(0.0)	1	(1.7)	1	(1.0)			
Clinical information at admission										
*Fazekas Scale*		0.0	[0.0, 1.5]	1.2	[0.0, 2.4]	1.0	[0.0, 2.5]		.104	.070
*Duration from ictus to admission (h)*		12.0	[4.5, 48.0]	6.0	[2.5, 10.0]	9.5	[2.0, 35.0]		.152	.417
*NIHSS score at admission*		1	[0, 2]	0	[0, 1]	2	[0, 4]		.002*	.050*
*NIHSS score day 2*		0	[0, 1]	0	[0, 0]	0	[0, 3]		.004*	.005*
*Systolic blood pressure (mmHg)*		158.5	[137.0, 173.2]	166.5	[145.0, 187.0]	164	[142.5, 185.5]		.323	.258
*Diastolic blood pressure (mmHg)*		83	[77.0, 89.0]	84.5	[74.8, 90.0]	86.5	[78.0, 94.8]		.845	.297
*Temperature (celcius)*		36.5	[36.2, 37.1]	36.6	[36.3, 36.8]	36.6	[36.2, 36.8]		.818	.999
*Haemoglobin (g/dL)*		14	[13.4, 14.8]	14.1	[12.9, 14.9]	14.2	[13.3, 15.1]		.999	.979
*eGFR*		89	[79.1, 99.8]	78	[63.0, 89.2]	83.1	[61.3, 93.8]		.059	.184
*ALT (U/L)*		34.5	[27.5, 48.2]	28.5	[21.2, 33.8]	27	[23.0, 33.0]		.004*	.003*
*Glucose (mmol/L)*		5.4	[5.0, 6.2]	5.5	[5.2, 6.3]	6.1	[5.5, 8.3]		.818	.019*
*LDL (mmol/L)*		2.6	[1.6, 3.5]	2.3	[1.6, 3.0]	2.5	[1.8, 3.3]		.456	.735
Blood-based biomarkers										
*Brain-derived tau (pg/mL)*		10.13	[7.8, 15.55]	12.36	[9.48, 16.99]	16.42	[10.08, 24.56]		.126	.004*
*Phosphorylated-tau 217 (pg/mL)*		7.50	[3.39, 11.27]	7.71	[4.63, 12.47]	7.76	[3.65, 13.04]		.999	.968
*Neurofilament light chain (pg/mL)*		65.94	[37.69, 110.39]	125.11	[65.36, 199.50]	145.93	[84.68, 329.01]		<.001*	.011*

*Denotes the p-values at a significance level of <0.05.

Results presented as median [IQR] unless otherwise stated. n: number of observations; TIA: transient ischemic attack; AIS: acute ischemic stroke; h: hours; NIHSS: National Institutes of Health Stroke Scale; eGFR: estimated glumerular filtration rate; ALT: alanine aminotransferase; LDL: low-density lipoprotein.

### BD-tau correlates with cortical stroke diameter

Inpatient MRI scans were obtained in 66 (64.7%) of AIS patients. Based on these scans, 36 (54.5%) patients were diagnosed with cortical stroke and 30 (45.5%) were diagnosed with subcortical stroke. Cortical stroke diameter exhibited strong correlation with BD-tau (tau-b = .64, p < .001) and p-tau217 (tau-b = .36, p < .01), but not with NfL (p = .163). Conversely, subcortical stroke diameter did not correlate with any of the biomarkers (Figure 1). This was further explored in a backwards stepwise linear regression, which confirmed a strong association with BD-tau as the single strongest explanatory variable for stroke diameter (Figure 1). None of the other eight independent variables entered the equation.

Among those that underwent an MRI scan, WML were present in 66.7% of AIS, 56.7% of TIA and 32.0% of the mimics. Distribution of Fazekas scale trended towards higher values in TIA and AIS patients (p = .060). Overall, Fazekas scale was strongly correlated with age (tau-b = .529, p < .001). Furthermore, we observed correlations with BD-tau (tau-b = .243, p < .001), p-tau217 (tau-b = .344, p < .001) and NfL (tau-b = .354, p < .001).

## Discussion

Early confirmation of an AIS is important for acute treatment and clinical management. This study aims to explore the clinical value of plasma biomarkers in the setting of an acute stroke ward. All patients with suspected AIS were non-selectively admitted to the stroke ward, resulting in inclusion of patients with other diagnoses than AIS. In our cohort, 16.1% of admitted patients were diagnosed as stroke mimics. This incidence of mimics is lower than that reported in a recent review (23.2%),^4^ which may be explained by improved pre-hospital diagnosis and management.

Plasma concentrations of BD-tau and NfL were significantly higher in AIS patients compared to stroke mimics, whereas p-tau217 was not different across the groups. It is worth emphasizing that p-tau217 in particular is strongly associated with tauopathy in AD,^23,24^ which none of the patients in the cohort were diagnosed with. The observed difference among these markers in differentiating AIS from mimics may suggest that despite sharing some similarities, their dynamic in blood reflect different pathophysiological aspects of cell injury and cell death.

Moreover, plasma BD-tau was strongly correlated with the stroke diameter as measured on an MRI scan. This finding corroborates the CNS origin of this marker, and suggests that similar to CSF total tau,^25^ increased levels of plasma BD-tau in stroke are indicative of the severity of the acute neuronal injury. Interestingly, the correlation between stroke diameter and tau was the strongest in cases of cortical stroke, whereas subcortical strokes showed no linear correlation. Because tau, in particular p-tau217, is reflective of synaptic pathology in AD,^26,27^ the strong correlation between tau release and stroke diameter could be explained by cortical synaptic structures^14^ being particularly susceptible to injury in AIS. Alternatively, subcortical strokes, which are typically of small vessel disease aetiology, are usually smaller and of less varying size than cortical strokes, which complicates establishing a clear linear association. Of note, none of the biomarkers were strongly correlated with the clinical NIHSS score at admission, whereas a strong correlation has been reported in previous studies.^28^

Although the diagnostic potential of tau biomarkers must be further explored, our findings support the potential role of BD-tau as an in-hospital biomarker to aid clinical diagnosis. Additionally, tau markers may have a role in predicting long-term function, as suggested by the significant association to the stroke diameter. Our findings are in agreement with other published studies on biomarkers for AIS. Bitsch et al. published the first prospective study^29^ demonstrating that the increase and duration of increase of plasma tau was correlated stroke size on MRI and physical disability at 3 months (assessed with modified ranking scale). Similarly, studies have demonstrated an association between high tau values and worse clinical outcome after stroke.^19,30,31^ The increase in tau is usually attributed to neuronal cell death during and after infarction. Post-mortem assessment demonstrated increase in tau immunoreactivity and deposition of tau in microglia^32^ and oligodendrocytes^33^ in the ischemic area possibly due to catabolic and degenerative processes.

The main limitations of this study were that biomarker quantification was limited to the day after admission. A recent study showed that food intake prior to blood sampling significantly altered the values of tau,^34^ something we unfortunately were unaware of at the time of this study, and therefore are unable to correct for. Moreover, tau proteins are shown to peak 3–5 days after AIS,^29^ while NfL peaks weeks after acute brain injury,^18^ thereby presumably underestimating the values in our dataset. Additionally, MRI scans were not available for all patients; however all patients underwent detailed clinical examination and CT scans. Subgroup analysis of thrombolysis or thrombectomy was not performed due to the small sample size of patients that underwent recanalization treatment. Another limitation is that we lacked detailed information on cognitive status and potential amyloid pathology in this cohort. Although none of our patients had established AD, it is possible that some had preclinical dementia or tauopathies, which could influence our findings. Future studies should address these questions in larger cohorts.

## Conclusions

In conclusion, our study demonstrates that plasma tau, in particular BD-tau, may be a promising biomarker for differentiating AIS from stroke mimics in the acute setting. Moreover, we found a strong correlation between BD-tau and cortical stroke diameter. The prognostic potential of BD-tau in AIS patients should be further evaluated in a future study.

## Supplemental Material

sj-pdf-1-jcb-10.1177_0271678X241293537 - Supplemental material for Neuronal plasma biomarkers in acute ischemic stroke Supplemental material, sj-pdf-1-jcb-10.1177_0271678X241293537 for Neuronal plasma biomarkers in acute ischemic stroke by Julia K Gundersen, Fernando Gonzalez-Ortiz, Thomas Karikari, Bjørn-Eivind Kirsebom, Katrin Mertes, Henrik Zetterberg, Hlin Kvartsberg, Ole Morten Rønning, Berglind Gísladóttir, Kaj Blennow and Tormod Fladby in Journal of Cerebral Blood Flow & Metabolism
