# Supplementary material for: Neuronal plasma biomarkers in acute ischemic stroke
Source: J Cereb Blood Flow Metab. 2024 Oct 25;45(1):77–84. doi: 10.1177/0271678X241293537 (PMC11563507; doi:10.1177/0271678X241293537)
Supplement: sj-pdf-1-jcb-10.1177_0271678X241293537 - Supplemental material for Neuronal plasma biomarkers in acute ischemic stroke [file sj-pdf-1-jcb-10.1177_0271678X241293537.pdf]

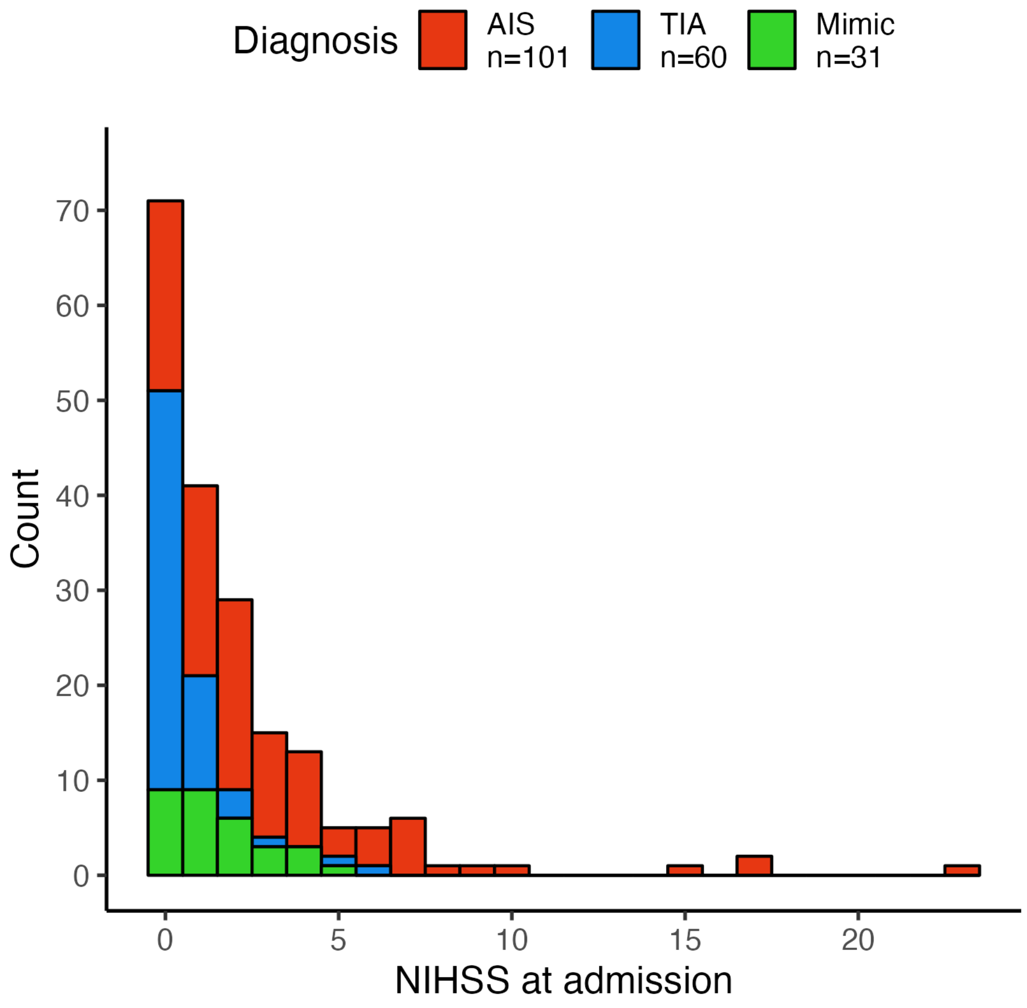

Supplementary figure 1. Distribution of National Institutes of Health Stroke Scale (NIHSS) at admission stratified by diagnosis: acute ischemic stroke (AIS), transient ischemic attack (TIA) or stroke mimic. Generally, NIHSS scores were skewed towards lower values, with a few outliers representing patients with AIS with high symptom burden.
